# A Bioluminescence Resonance Energy Transfer-Based Approach for Determining Antibody-Receptor Occupancy *In Vivo*

**DOI:** 10.1016/j.isci.2019.05.003

**Published:** 2019-05-08

**Authors:** Yu Tang, Kshitij Parag-Sharma, Antonio L. Amelio, Yanguang Cao

**Affiliations:** 1 Division of Pharmacotherapy and Experimental Therapeutics, UNC Eshelman School of Pharmacy, University of North Carolina at Chapel Hill, 2318 Kerr Hall, Chapel Hill, NC 27599-7569, USA; 2 Graduate Curriculum in Cell Biology & Physiology, Biological & Biomedical Sciences Program, School of Medicine, University of North Carolina at Chapel Hill, Chapel Hill, NC 27599, USA; 3 Biomedical Research Imaging Center, School of Medicine, University of North Carolina at Chapel Hill, Chapel Hill, NC 27599, USA; 4 Lineberger Comprehensive Cancer Center, School of Medicine, University of North Carolina at Chapel Hill, Chapel Hill, NC 27599, USA

**Keywords:** Optical Imaging, Biological Sciences, Cell Biology

## Abstract

Elucidating receptor occupancy (RO) of monoclonal antibodies (mAbs) is a crucial step in characterizing the therapeutic efficacy of mAbs. However, the *in vivo* assessment of RO, particularly within peripheral tissues, is greatly limited by current technologies. In the present study, we developed a bioluminescence resonance energy transfer (BRET)-based system that leverages the large signal:noise ratio and stringent energy donor-acceptor distance dependency to measure antibody RO in a highly selective and temporal fashion. This versatile and minimally invasive system enables longitudinal monitoring of the *in vivo* antibody-receptor engagement over several days. As a proof of principle, we quantified cetuximab-epidermal growth factor receptor binding kinetics using this system and assessed cetuximab RO in a tumor xenograft model. Incomplete ROs were observed, even at a supratherapeutic dose of 50 mg/kg, indicating that fractional target accessibility is achieved. The BRET-based imaging approach enables quantification of antibody *in vivo* RO and provides critical information required to optimize therapeutic mAb efficacy.

## Highlights

• Nano-BRET was used to longitudinally quantify cetuximab-binding kinetics to EGFR
• Incomplete EGFR occupancy in solid tumors was observed even at supratherapeutic doses
• A kinetic disassociation exists between plasma antibody and bound targets in tumors

## Graphical Abstract

**Figure:**
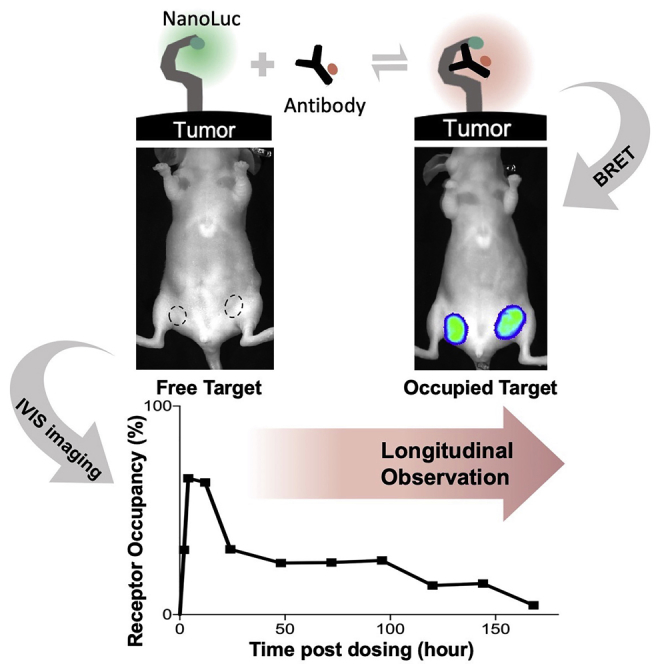

## Introduction

Monoclonal antibodies (mAbs) are often regarded as “magic bullets” (Brodsky, 1988), which have been applied toward the treatment of an array of human diseases (Mould and Sweeney, 2007). These therapeutic mAbs are engineered to specifically bind their cognate antigens with high affinities and have been deployed for neutralizing pathologic factors, blocking cellular signaling, and stimulating immune functions (Suzuki et al., 2015). Therapeutic mAbs have shown great promise in cancer treatments given their therapeutically desirable characteristics of long plasma half-lives, high selectivity, and limited off-target toxicity (Wang et al., 2008). To date, over 30 mAbs (and rising) have been approved for treatment of various types of cancers, including hematologic malignancies and solid tumors (Reichert, 2012, Reichert, 2016, Reichert, 2017, Ecker et al., 2015, Kaplon and Reichert, 2018).

Like other targeted therapies, mAbs can only elicit their desired pharmacological effects when directly bound to their cognate targets. Therefore elucidating the target engagement of a given mAb is a crucial step toward characterizing its therapeutic potential and in determining its pharmacological dynamics, which helps define the optimal dosing regimens to achieve maximal therapeutic efficacy. Target engagement, or receptor occupancy (RO), is the ratio of occupied receptors of interest over total receptors of interest present on the targeted cells. Establishing the RO profile of any therapeutic mAb via preclinical or clinical studies is critical toward projecting the first-in-human dosages, to ensure minimal anticipated biological effect level and minimize potential dose-limiting toxicity (Agoram, 2009, van Gerven and Bonelli, 2018; Duff, 2006). Antibody RO is often a valuable intermediate measurement for establishing dose (or exposure)-response relationships, especially at early stages of mAb development when defined biomarkers for an mAb's pharmacological effects are not available (Agoram, 2009, Liang et al., 2016, Shi et al., 2017). Although many other factors should be considered when interpreting RO, such as receptor epitope properties (Lipman et al., 2005, Rook et al., 2015), antibody-receptor binding is the first step required to elicit a pharmacological effect, and the binding kinetics of a given mAb to its targets within the tumor microenvironment dictates its general therapeutic potential.

Tremendous efforts have been expended toward creating a reliable and cost-effective method to quantify antibody RO. Flow cytometry (FCM), owing to its ease of operation, is routinely used to determine RO (Topalian et al., 2012, Liang et al., 2016); however, FCM is only ideally suited to antibodies that have targets present on circulating blood cells. Moreover, the constraints on sampling accessibility and high spatial heterogeneity often hinder the use of FCM toward antibodies targeting peripheral tissues. Large disparities have been observed between antibody concentrations in circulating plasma and in solid tumors (Suh et al., 2016, Bartelink et al., 2018). Owing to the large sizes, high binding affinities, and high target specificities (Weinstein et al., 1987), the distribution of antibodies in dense interstitial matrix is often limited to the perivascular area, resulting in fractional accessibility of targets to mAbs. In solid tumors, antibody-target binding kinetics and the resultant RO are subject to complex biological variables, including tumor-blood perfusion, antibody extravasation across the tumor vasculature, tumor extracellular matrix densities, and the expression levels and accessibility of antigens on tumor cells that are recognized by mAbs. All these factors complicate reproducibly quantifying antibody-target binding kinetics and the resultant RO in solid tumors.

One approach to quantify antibody RO in solid tumors is to perform immunohistochemistry staining on tumor biopsies. However, this approach lacks temporal resolution and often fails to incorporate dynamic factors present in *in vivo* situations that could greatly influence mAb-target interactions (Gebhart et al., 2016). Another approach to assess antibody RO within solid tumors is to perform radiotracer replacement studies, which usually require two steps: first, giving subjects a small dose of radiolabeled antibody, and then giving increasing doses of unlabeled antibody. Owing to competitive binding, the radioactivity levels in the tumors decrease as doses of unlabeled antibody increase, indicating an increased RO, until a plateau is gradually achieved. Determining mAb RO using this approach is often complicated by the rapid endocytosis of the radiotracers by tumors (Boswell et al., 2010). The estimation of RO is further biased by the unstable radioactivity in the control group, which should have relatively constant radioactivity without competitive replacement by unlabeled antibodies (Cunningham et al., 2005).

Other radiolabeling methods, including positron emission tomography/single-photon emission computed tomography, are often applied to quantify mAb pharmacokinetics (PK), tissue distribution, and tissue-specific RO. These approaches raise safety concerns when determining the mAb RO due to elevated radiation accumulation (Burvenich et al., 2018). Fluorescence imaging has also been explored for both preclinical and clinical applications (Rosenthal et al., 2015, Warram et al., 2015, Saccomano et al., 2016, Lamberts et al., 2017, Fornasier et al., 2018, Miller et al., 2018). However, fluorescent imaging suffers from fluorescence quenching that is caused by external excitation light, and poor signal:noise ratios due to the high autofluorescence of biological tissues. Aside from these intrinsic disadvantages, most current non-invasive *in vivo* imaging methods have a common drawback to RO quantification, namely, they are unable to distinguish signals arising due to specific target engagement versus non-specific background signals. At the tissue level, it is difficult to distinguish the signals of bound mAbs from those of free mAbs present in blood circulating within tissues. Probes or tracers can exhibit non-specific binding and residualization in tumors, which greatly bias RO quantification (Cunningham et al., 2005, Patel and Gibson, 2008, Ogawa et al., 2009). Therefore a non-invasive imaging technology that exclusively enables the visualization of antibody-target interactions *in vivo* is greatly desired.

In the present study, we developed a bioluminescence resonance energy transfer (BRET)-based system to non-invasively quantify antibody RO in live animals. BRET detection schemes are based on Förster resonance energy transfer, in which resonance energy is transmitted from a luciferase molecule (donor) during substrate catalysis to a fluorescent molecule (acceptor), which then re-emits the light according to its own emission spectra (Dragulescu-Andrasi et al., 2011). In BRET-based protein-protein interaction studies, the donor (luciferases) and acceptor (fluorophores) molecules are tagged onto two distinct proteins of interest. Interaction between the proteins of interest, upon appropriate stimuli, brings the luciferase and fluorophore into close proximity, enabling the luciferase to efficiently transmit energy to the fluorophore resulting in BRET (Mandic et al., 2014, Ciruela and Fernandez-Duenas, 2015, Machleidt et al., 2015, Coriano et al., 2016, Goyet et al., 2016, Alcobia et al., 2018, Rathod et al., 2018). BRET efficiency is governed by both the distance and orientation of the donor and acceptor molecules relative to each other. Given the stringent requirements of distance separation (∼10 nm) between donor-acceptor molecules for efficient BRET, it offers a large signal:noise ratio and high sensitivity at physiologically relevant temporal resolutions and therefore has found wide utility in ligand-target interaction studies (Machleidt et al., 2015, Mo and Fu, 2016). Recently, BRET imaging was applied to visualize a propranolol-dye conjugate (acceptor-ligand) binding to an N-terminal NanoLuc (NLuc)-tagged human G-protein-coupled receptor *β*_2_-adrenoreceptor (donor-receptor) in real time (Alcobia et al., 2018), demonstrating the noteworthy performance of BRET imaging for monitoring ligand-receptor binding *in vivo*. In the present study, we extended the BRET approach to clinically attractive mAb therapies. Cetuximab (CTX), a therapeutic mAb currently deployed in many clinical trials for solid tumors, and its cognate target receptor, epidermal growth factor receptor (EGFR), were selected as a model system. EGFR is one of the most well-studied receptors; mediates key growth factor response pathways driving cell survival, proliferation, and growth; and has been implicated (overexpression/mutations) in numerous human malignancies (Han and Lo, 2012, Seshacharyulu et al., 2012, Ceresa and Peterson, 2014, Song et al., 2016, Liu et al., 2017, Sigismund et al., 2018). Herein, we present a BRET-based imaging approach to directly monitor the temporal profiles of antibody-target RO in live animals using CTX binding to EGFR.

## Results

### Design of the NanoLuc-EGFR/DY605-Cetuximab BRET Imaging System

**Figure 1:**
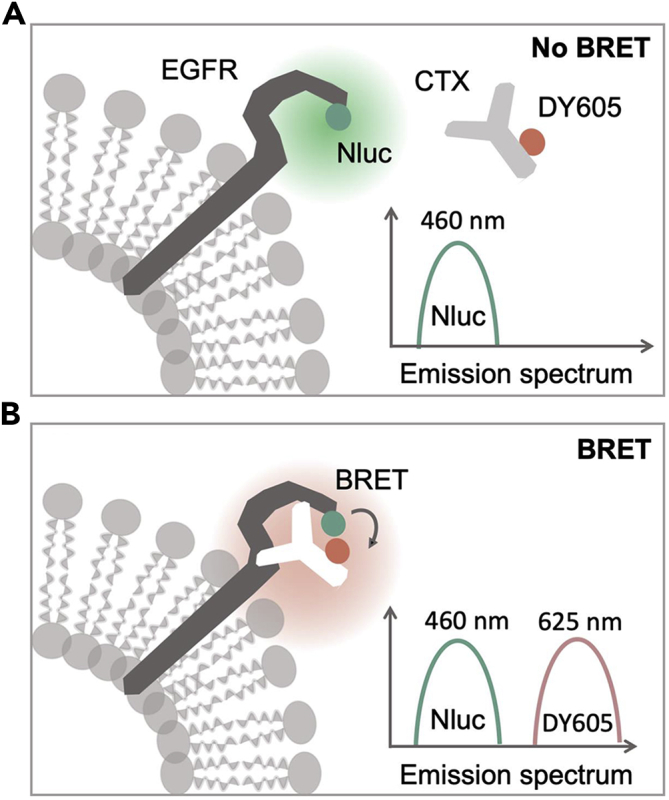
Schematic of the NanoLuc-EGFR/DY605-Cetuximab BRET System NanoLuc (a 19-kDa luciferase) was fused to the N terminus of the epidermal growth factor receptor (EGFR) extracellular domain to generate the BRET donor moiety. A fluorescent dye, DY605, was covalently appended onto cetuximab to generate the BRET acceptor moiety. (A) In the absence of DY605-cetuximab binding, only the NanoLuc-EGFR fusion donor emission at 460 nm was detected upon addition of the substrate (furimazine), because the distance between unbound acceptor and donor moieties is too large to trigger BRET. (B) In the presence of DY605-cetuximab and upon its target engagement with the NanoLuc-EGFR fusion, furimazine administration generates two distinct emission peaks, at 460 nm (NanoLuc) and 625 nm, the latter arising from DY605-cetuximab as a result of the robust BRET between NanoLuc and DY605, now brought into close proximity by the NanoLuc-EGFR:DY605-cetuximab interaction. Donor (at 460 nm) and acceptor (at 625 nm) emission peaks are ∼165 nm apart ensuring robust spectral separation and reliable detection.

NLuc (Hall et al., 2012), which has shown significant advantages over other luciferases in BRET-based studies, such as enhanced maximal light output, improved signal stability, ATP-independent light generation, and resistance to autoinhibition among others (Schaub et al., 2015, England et al., 2016, Alcobia et al., 2018), was fused to the N terminus of EGFR to generate the NLuc-EGFR fusion protein (Figure S1A). A fluorescent dye, DY605, was covalently labeled onto CTX to generate the DY605-CTX conjugate (DY605-CTX). In the absence of DY605-CTX binding, the addition of the NLuc substrate (furimazine) results in the single emission peak at 460 nm (NLuc, the BRET donor) (Figure 1A). However, upon DY605-CTX binding to NLuc-EGFR (bringing NLuc into close proximity with the DY605 fluorophore), the addition of furimazine produces two distinct peaks at 460 and 625 nm (emission peak of DY605, the BRET acceptor), the latter arising from the robust BRET observed between NLuc and DY605 (Figure 1B). Donor (at 460 nm) and acceptor (at 625 nm) emission peaks are ∼165 nm apart ensuring robust spectral separation and reliable detection.

### Characterization of the BRET System

**Figure 2:**
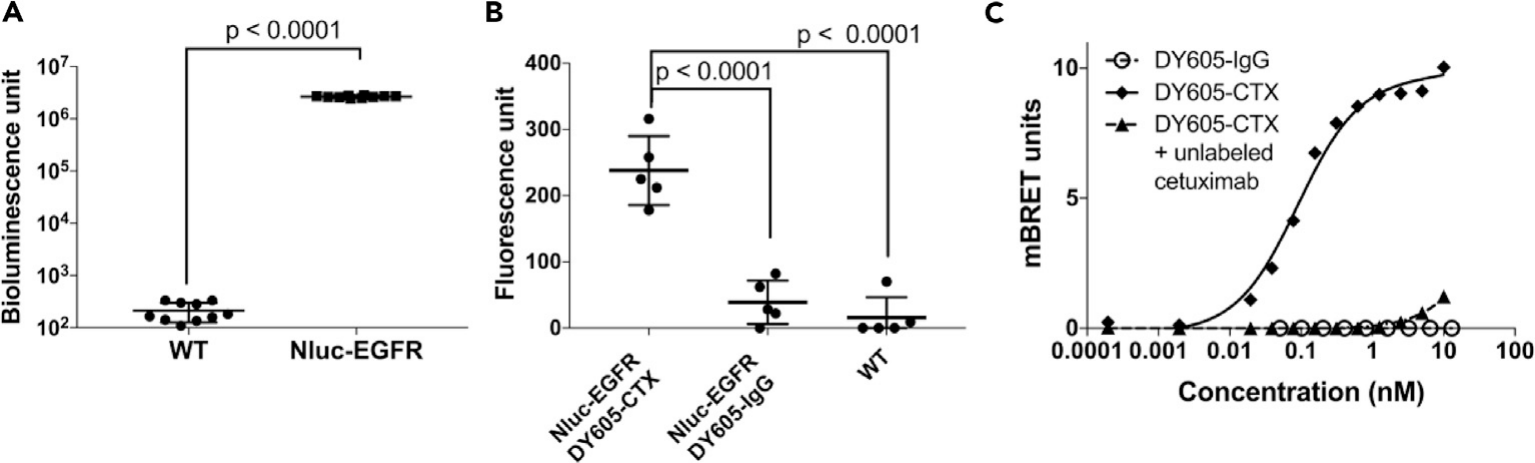
Generation and Characterization of Stable NanoLuc-EGFR HEK293 Cells (A) Under identical conditions, the BRET donor (NanoLuc) bioluminescent signal of NanoLuc-EGFR HEK293-stable cells was ∼10,000 fold higher compared with wild-type HEK293 cells (two tailed, unpaired Students *t* test, p < 0.0001). (B) EGFR fusion expression levels were probed using DY605-cetuximab and found to be ∼5-fold higher in the NanoLuc-EGFR stable cells compared with wild-type HEK293 cells (two tailed, unpaired Students *t* test, p < 0.0001). For both panels (A) and (B), representative results are shown. Each data point represents one technical replicate. Error bars represent ±SD values. At least three independent biologic replicates were performed per experiment. (C) BRET activity from the NanoLuc-EGFR/DY605-cetuximab system is concentration dependent (K_D_ = 0.1 ± 0.01 nM, B_max_ = 9.9 ± 0.24 mBRET units). No BRET was observed for the DY605-IgG control indicating negligible non-specific binding. Moreover, DY605-cetuximab binding to NanoLuc-EGFR is highly specific because no BRET signal was detected in the group containing DY605-cetuximab + unlabeled cetuximab (1 mM) due to the competition for the same EGFR domain. Each data point represents the mean value of three technical replicates. Data are presented as mean ± SD. The experimental results are representative of at least three biologic replicates. For panels (B and C), the DY605-cetuximab has DAR = 3.8.

To characterize NLuc-EGFR expression in our HEK293 cells stably expressing NLuc-EGFR, we measured both NLuc and EGFR expression levels using bioluminescence signal intensity and anti-EGFR antibody fluorescence, respectively. As expected, the NLuc signal in the NLuc-EGFR HEK293 cells is several orders of magnitude higher than the background signal in the parental wild-type (WT) HEK293 cells (Figure 2A). Antibody probing for EGFR revealed a robust upregulation (5-fold) of EGFR protein levels in the NLuc-EGFR HEK293 cells compared with the parental WT HEK293 cells (Figure 2B).

Next, we characterized the DY605-CTX binding affinity to NLuc-EGFR. DY605-IgG was used as a negative control, and binding specificity of DY605-CTX to NLuc-EGFR was evaluated using a competition assay in the presence of a saturating concentration of unlabeled CTX (1 mM). The saturation binding curves (Figure 2C) suggested single-site binding of DY605-CTX to NLuc-EGFR with an affinity of K_D_ = 0.1 ± 0.01 nM, which is in agreement with previously reported K_D_ for CTX-EGFR binding (0.39 nM) (Kim and Grothey, 2008). The binding of DY605-CTX to NLuc-EGFR could be completely blocked by a high concentration of unlabeled CTX, suggesting that DY605-CTX retains its site specificity toward EGFR, compared with unmodified CTX. DY605-IgG exhibited no non-specific binding to NLuc-EGFR. Collectively, the BRET signal observed from interaction between DY605-CTX and NLuc-EGFR could be used to quantify its binding kinetics and to evaluate the degree of RO between CTX and EGFR.

### Effect of Dye per Antibody Ratio on Binding Affinity and Potentially Altered PK of DY605-Cetuximab in Mice

**Figure 3:**
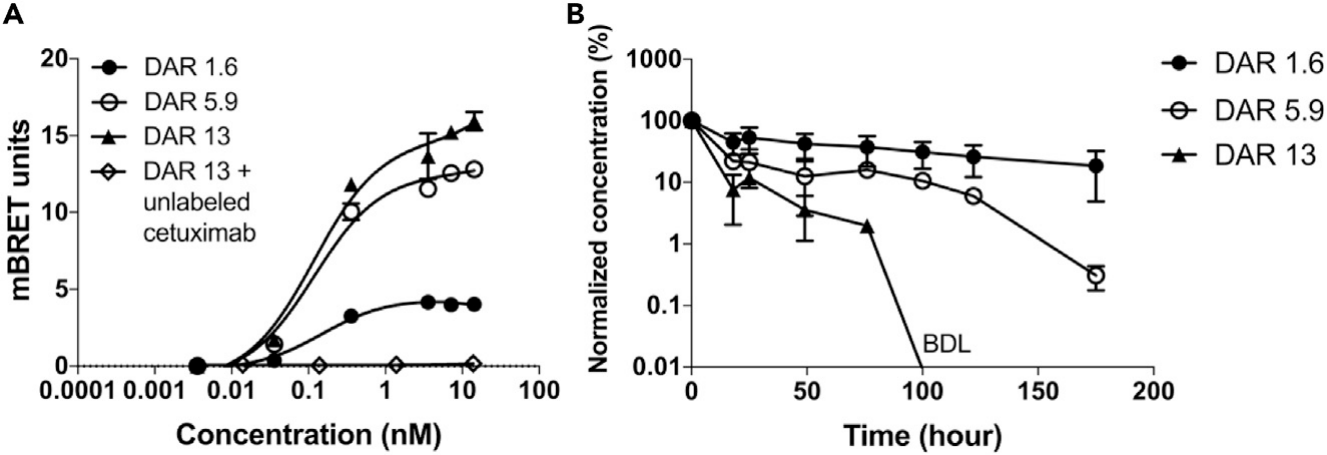
Evaluating the Effects of Dye-Antibody Ratios (DAR) on *In Vitro* Binding Affinity and *In Vivo* Pharmacokinetics (A) The binding affinities of DY605-cetuximab to NanoLuc-EGFR at various dye per antibody ratios (DARs) = 1.6, 5.9, and 13 were similar (0.15 ± 0.04, 0.12 ± 0.03, and 0.12 ± 0.03, respectively), whereas the maximum BRET signals increased (4.8 ± 0.3, 13.4 ± 0.6, and 15.6 ± 0.8, respectively). (B) High DAR is correlated with increased DY605-cetuximab clearance *in vivo*. Plasma concentrations were normalized to initial concentrations. Plasma concentrations below the detection limits were noted as BDL. For panels (A and B), each data point represents the mean value of three technical replicates. Data are presented as mean ± SD. BDL, below detection limit.

To determine the effect of dye per antibody ratio (DAR) on CTX's affinity to NLuc-EGFR, we generated DY605-labeled CTX at multiple DARs (1.6, 5.9, and 13). As shown in Figure 3A and Table 1, the K_D_ values of DY605-CTX to NLuc-EGFR remain consistent across a wide range of DARs (K_D_ = 0.15 ± 0.04, 0.12 ± 0.03, and 0.12 ± 0.03 nM at DAR = 1.6, 5.9, and 13, respectively). As expected, a greater maximal BRET signal (B_max_) was observed at higher DAR, B_max_ = 4.8 ± 0.3, 13.4 ± 0.6, and 15.7 ± 0.8 at DAR = 1.6, 5.9, and 13, respectively. Compared with DAR = 1.6, B_max_ at DAR = 5.9 increased ∼3-fold. However, B_max_ only increased ∼1.2-fold between DAR = 5.9 and DAR = 13 conditions (Figure 3A and Table 1), indicating that the BRET signals achieved are near saturation at the highest DAR tested herein.

**Table 1:** Binding Constants and Pharmacokinetic Parameters of DY605-CTX Conjugate with Different DARs

Groups	Binding Parameters		PK Parameters		
	B_max_ (mBRET)	K_D_ (nmol/L)	C_max_ (nmol/L)	AUC (nmol⋅h/L)	Clearance (mL/h)
DAR 1.6	4.8 (0.3)	0.15 (0.04)	490 (202)	29,663 (11,471)	0.009 (0.005)
DAR 5.9	13.4 (0.6)	0.12 (0.03)	302 (40)	7,511 (3,462)	0.065 (0.039)
DAR 13	15.7 (0.8)	0.12 (0.03)	310 (12)	3,932 (522)	0.100 (0.014)

Data are expressed as mean (±SD).

CTX, cetuximab; DAR, dye per antibody ratio.

We also compared the PK of DY605-CTX at different DARs. Nude mice were injected with three DY605-CTX DAR variants (DAR = 1.6, 5.9, and 13) at 3.2 mg/kg via tail vein. Blood samples were collected at 0, 18, 24, 48, 72, 96, 120, and 168 h post dosing. As shown in Figure 3B, three DY605-CTX conjugates at the same dose level had significantly different systemic clearance. The PK parameters are summarized in Table 1. The DY605-CTX displaying the highest Bmax was DAR = 13, whereas DY605-CTX with DAR = 13 showed the fastest clearance (0.100 ± 0.014 mL/h), compared with 0.009 ± 0.005 and 0.065 ± 0.39 mL/h for labeled CTX at DAR = 1.6 and DAR = 5.9, respectively, and had concentration below detectable level by 96 h post-dosing. To maintain the BRET imaging efficiency while minimizing any DAR-associated alteration to antibody PK, we therefore aimed to label CTX at DAR that was close to 5.9 for subsequent RO assessment.

### Plasma Stability of DY605-CTX

To assess the plasma stability of the DY605-CTX and DY605-IgG conjugate (DAR = 4.6 and 5.6, respectively), we first confirmed that the residual free dye in the drug samples was negligible (Figure S2A, 3.6% for DY605-CTX and 3.3% for DY605-IgG). DY605-conjugated IgG and CTX were incubated in mouse plasma at 37°C for 5–9 days, following which their total fluorescent intensities were evaluated. Plasma incubation had no significant effect on the fluorescent intensities of either DY605-CTX or DY605-IgG conjugates (Figure S2B, p = 0.7), suggesting minimal fluorescence quenching. Throughout the incubation, no conjugate dissembling was detected in either DY605-CTX or DY605-IgG (Figure S2C). Thus DY605-CTX and DY605-IgG conjugates are stable in mouse plasma at body temperature over extended periods of time (up to 9 days).

### Development of NLuc-EGFR HEK293 Xenograft

**Figure 4:**
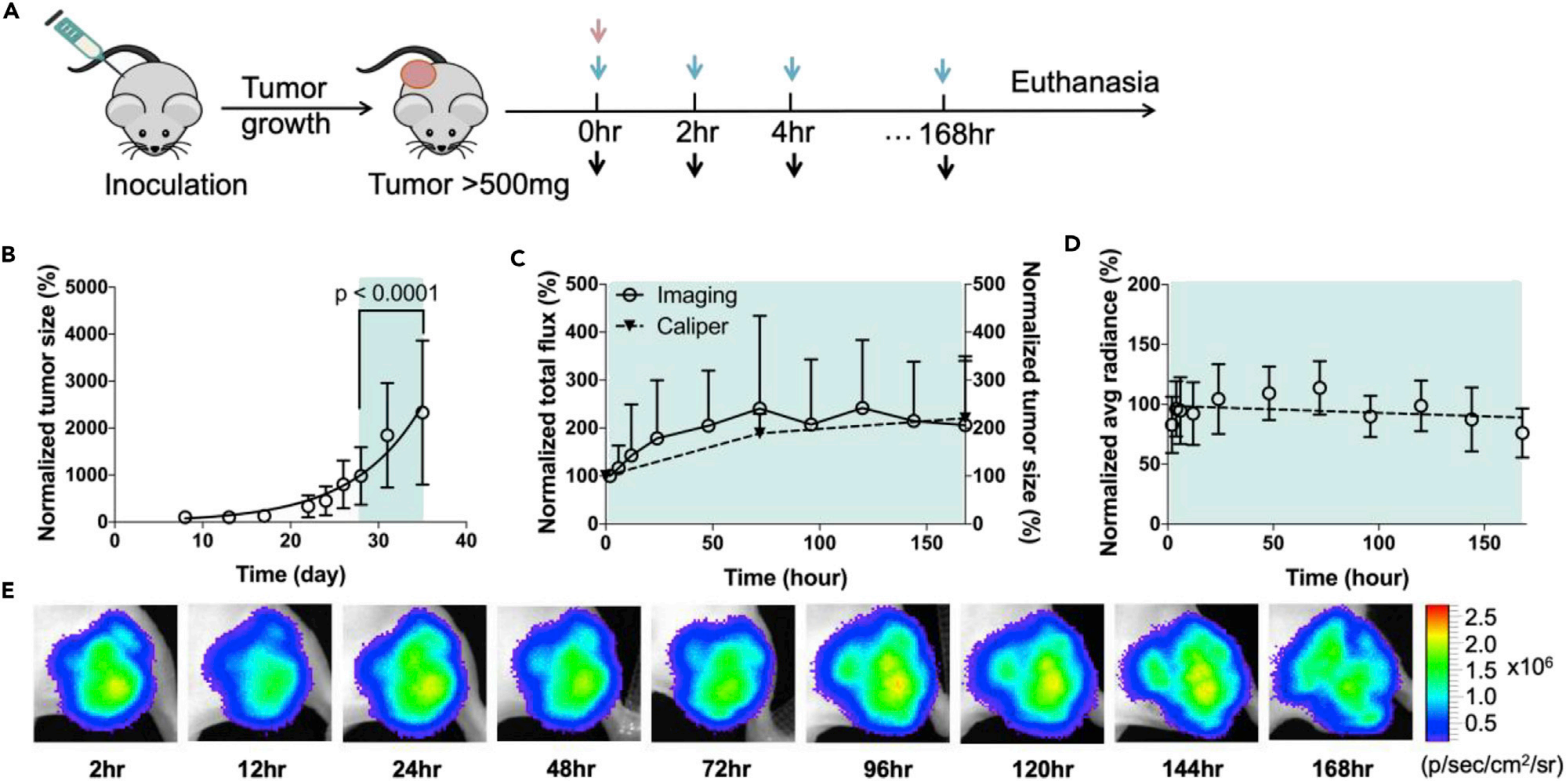
*In Vivo* Characterization of NanoLuc-EGFR Tumor Growth Kinetics (A) Schematic overview of the tumor inoculation, drug conjugate administration, IVIS bioluminescent imaging, and blood sample collection for animals administered DY605-antibody conjugates. Red arrow, tail vein injections of either DY605-cetuximab or DY605-IgG (control); blue arrows, tail vein injections of the NanoLuc substrate (furimazine); black arrows, imaging acquirements and blood sample collections. (B) Tumor growth curve from the day the tumor was inoculated (day 0) to day 35. The caliper-measured tumor sizes were normalized to the first-observed tumor volumes and fitted to the exponential growth curve. Normalized tumor volumes at days 28 and day 35 were compared using a two-tailed, unpaired Student's *t* test. (C) Tumor total photon flux at 500 ± 20 nm (NanoLuc emission) relative to caliper-measured tumor growth was normalized to the initial measurements at the first day of *in vivo* receptor occupancy (RO) study (day 28). The increase in total flux was tested by a null hypothesis, “the slope of linear regression of the dataset is significantly non-zero”. The null hypothesis was not rejected by a p value of 0.01. The trends of the normalized tumor-NanoLuc total photon flux and the normalized tumor volumes, measured by calipers, were compared using a null hypothesis, “the slopes of the linear regressions are same for all datasets.” The null hypothesis was rejected by a p value of 0.736. (D) The NanoLuc-EGFR tumor densities were estimated using normalized tumor average radiances. The trend of average radiances was evaluated by testing the null hypothesis that “the slope of linear regression of the dataset is significantly non-zero,” which was rejected by a p value of 0.34. During the *in vivo* RO detection phase of the study, NanoLuc-EGFR densities did not change significantly. For panels (B–D), the RO detection phase of this *in vivo* study is highlighted by the green area (day 28 to day 35, 0–168 h post dosing). All the animals (n = 19) were included in the tumor size, tumor area total flux, and average radiance analyses. Each data point represents the mean value of the tumor size, tumor area total flux, and average radiance of 19 individuals at the same time point. Data are presented as mean ± SD. (E) Representative images of the tumor NanoLuc-EGFR densities (radiance intensity) measured throughout the *in vivo* RO phase of the study.

A schematic of the study design and sampling strategy for the *in vivo* experiments is summarized in Figure 4A. Tumor sizes were monitored every other day using calipers. Implanted tumors exhibited exponential growth during the observation period (Figures 4B and S3), and as expected, the normalized tumor sizes at the end of the *in vivo* RO study (day 35) are significantly greater than the ones at the beginning (day 28) (p < 0.0001). During the study period, tumor growth was also quantified using bioluminescence at the NLuc emission wavelength (collected using a 500/20-nm band-pass filter). The caliper-measured tumor sizes and the total bioluminescence photon flux at tumor area were normalized to the initial values. The increase in total flux was statistically significant during the study (p = 0.01), and the increase in NLuc luminescence closely matches the increase in overall tumor size as measured using calipers (p = 0.736) (Figure 4C). This close relationship between tumor size (caliper measurements) and relative NLuc luminescence (imaging) is expected, given that the total photon flux generated is directly dependent on the number of NLuc-EGFR molecules present in the tumor, which is determined by the number of tumor cells (tumor size). The receptor density was estimated using average radiance, which represents the photon flux per unit area and unit solid angle. The receptor densities did not change significantly over the course of this study (Figures 4D and 4E, p = 0.34).

### Measurement of RO in Live Mice

**Figure 5:**
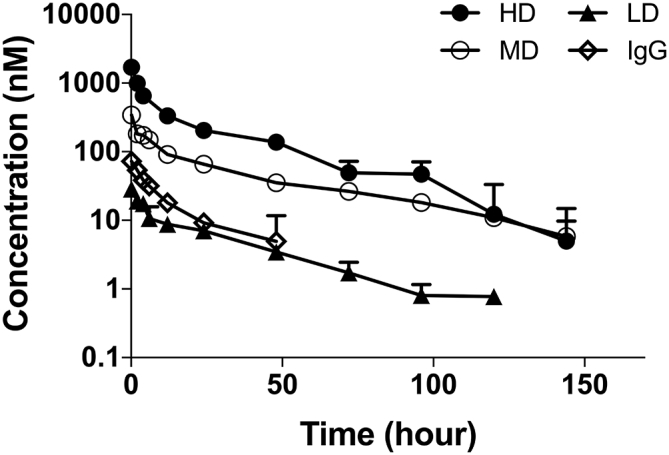
Establishing DY605-Cetuximab Pharmacokinetics *In Vivo* Profiles of mean (±SD) plasma concentrations versus time after a single tail vein injection of 1.0 (LD), 8.5 (MD), and 50 (HD) mg/kg DY605-cetuximab or 1.9 mg/kg DY605-IgG (control) in tumor-bearing animals. The decay phases of DY605-cetuximab and DY605-IgG PK curves exhibited a similar trend. Plasma concentrations of 50 mg/kg and 8.5 mg/kg groups were below detection limits at 144 h, whereas the plasma concentration of the 1 mg/kg group fell below detection limits at 120 h. The detection limits are 15, 5, 0.7, and 3 nM for HD, MD, LD, and control groups, respectively. Each data point represents the mean value of three replicates. Error bars represent ±SD. HD = High Dose (50 mg/kg); MD = Medium Dose (8.5 mg/kg); LD = Low Dose (1.0 mg/kg).

The RO assessment was initiated on day 28 and was continued until day 35, with a duration of 168 h (Figures 4B–4D). While monitoring DY605-CTX/NLuc-EGFR target engagement, we sought to establish the PK profile of DY605-CTX (DAR = 4.6) at multiple dosing paradigms: 50 mg/kg, 8.5 mg/kg, and 1.0 mg/kg. The PK curves revealed linear kinetics across all dosages tested (Figure 5). As shown in Table 2, the different doses had similar dose-normalized C_max_ (255 ± 17, 303 ± 32, and 214 ± 22 L^−1^, for 50 mg/kg, 8.5 mg/kg, and 1.0 mg/kg, respectively). The antibodies at 50 mg/kg and 1.0 mg/kg groups had similar dose-normalized area under the curve (AUC) (3,025 ± 453 and 3,814 ± 513 h/L, respectively). The antibodies at 8.5 mg/kg and 1.0 mg/kg groups showed similar clearance (0.19 ± 0.02 and 0.25 ± 0.03 mL/h, respectively). The dose-normalized AUC at 8.5 mg/kg was slightly higher than those of other two groups, but in general, the PK of DY605-CTX was linear within dose ranges of 1.0–50 mg/kg.

**Table 2:** Non-compartmental Analysis Results of the Pharmacokinetics DY605-CTX

PK Parameters	DY605-CTX 50 mg/kg	DY605-CTX 8.5 mg/kg	DY605-CTX 1.0 mg/kg	DY605-IgG 1.9 mg/kg
Cmax (nmol/L)	1,700 (115)	344 (36)	29 (3)	72 (11)
AUC (nmol·h/L)	20,176 (3,018)	5,917 (740)	508 (68)	804 (74)
Clearance (mL/h)	0.32 (0.04)	0.19 (0.02)	0.25 (0.03)	0.27 (0.04)
C_max_/dose (L^−1^)	255 (17)	303 (32)	214 (22)	286 (44)
AUC/dose (h/L)	3,025 (453)	5,222 (653)	3,814 (513)	3,176 (293)

Data are expressed as mean (±SD).

CTX, cetuximab.

**Figure 6:**
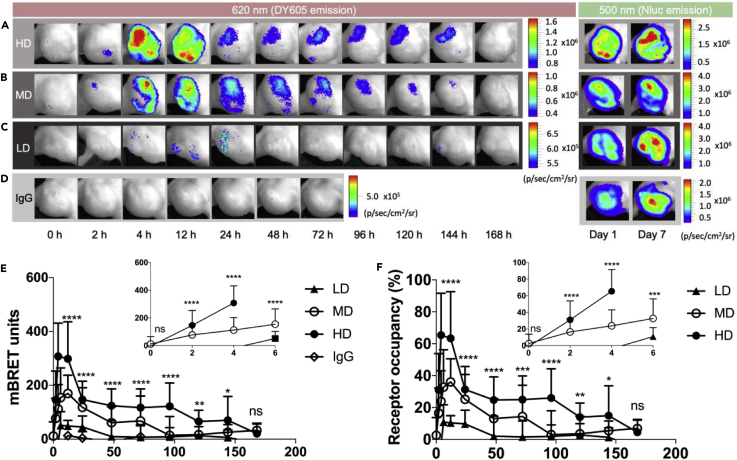
DY605-Cetuximab/NanoLuc-EGFR Receptor Occupancy (RO) Measurement *In Vivo* (A) BRET images (at 620 nm) of mice that received 50 mg/kg (HD) of DY605-cetuximab. The BRET measurement of HD group at 6 h post administration was absent due to a problem in data collection. (B) BRET images (at 620 nm) of mice that received 8.5 mg/kg (MD) of DY605-cetuximab. (C) BRET images (at 620 nm) of mice that received 1.0 mg/kg (LD) of DY605-cetuximab. (D) BRET images (at 620 nm) of control group mice. The mice in control group received 1.9 mg/kg of DY605-IgG. DY605-IgG showed no binding with NLuc-EGFR during the study. For panels (A–D), the representative NanoLuc images (at 500 nm) of each dose group at day 1 (the first day of *in vivo* RO study) and day 7 (the last day of *in vivo* RO study) are shown on the right. (E) Quantified NanoLuc-EGFR/DY605-cetuximab binding for the 1.0 mg/kg (LD), 8.5 mg/kg (MD), and 50 mg/kg (HD) groups and 1.9 mg/kg DY605-IgG (control). For HD, MD, and LD groups, the bindings peaked at about 4–12 h and experienced biphasic decay after 12 h post injection. The BRET ratios for 1.0 mg/kg group were compared to DY605-IgG group by a two tailed, unparied Student *t* test. The BRET ratios for 1.0 mg/kg group was not significantly different with IgG controls at 48 h post injection (p = 0.23). No binding was observed for DY605-IgG. (F) Quantified RO in live mice. Quantification of DY605-CTX RO revealed a maximum average RO for the HD, MD, and LD groups of 72% ± 26%, 41% ± 14%, and 18% ± 6%, respectively. For (E and F), each data point represents the mean value of at least 15 unique ROIs (n = 2–6 per mice). Error bars represent ±SD. The difference in RO across groups was evaluated using ordinary one-way ANOVA. ****p ≤ 0.0001, ***p ≤ 0.001, **p ≤ 0.01, *p ≤ 0.05, ns: p > 0.05.

Next the RO was quantified by BRET ratios, as described in the Transparent Methods (Equations 5 and 6). As expected, due to intratumoral heterogeneity, the BRET signal intensities were not homogeneous across the entire tumor area (Figures 6A–6C). To prevent RO measurement biases arising from tumor-intrinsic factors (intra- or intertumoral heterogeneity), we quantified the average gated region of interest (ROI) BRET ratios across all animals in any given dosage group, accounting for both intratumoral heterogeneity and intertumoral variances. The BRET signal of the control group (DY605-IgG) was negligible throughout the observation period (Figures 6D and 6E). In addition, the linear regression slope of DY605-IgG raw BRET ratios versus time data is not significantly different from zero (p = 0.58), suggesting negligible non-specific interactions between IgG and EGFR, further highlighting the robust signal:noise ratio offered by our BRET-based imaging approach. A time-dependent increase in BRET signal (DY605-CTX/NLuc-EGFR binding) was observed, suggesting that most of the DY605-CTX was still in the vascular space immediately post administration with increasing tumor penetration and target engagement over time (Figures 6A–6C and 6E). Across all doses of DY605-CTX, the BRET signal (and thus maximal DY605-CTX target engagement) observed gradually increased and peaked at ∼4 h post administration (Figures 6A–6C and 6E). Times of maximal target engagement were consistent across all doses tested. The maximum BRET ratios observed for 50, 8.5, and 1.0 mg/kg doses were 337 ± 123, 194 ± 76, and 77 ± 39 mBRET units, respectively (Table 3).

**Table 3:** Non-compartmental Analysis Results of Quantified Target Binding and Receptor Occupancy (RO)

Dose Groups	Binding Parameters			Receptor Occupancy Parameters		
	B_max_ (mBRET)	AUC_Binding_ (mBRET·h)	λ_Z-Binding_	RO_max_ (%)	AUC_RO_ (%·h)	λ_Z-RO_
50 mg/kg	337 (123)	19,411 (6,203)	0.011 (0.002)	72 (26)	4,083 (1,316)	0.01 (0.002)
8.5 mg/kg	194 (76)	8,406 (5,429)	0.013 (0.006)	41 (14)	2,244 (1,053)	0.01 (0.006)
1.0 mg/kg	77 (39)	2,272 (1,985)	0.010 (0.011)	18 (6)	855 (347)	0.01 (0.008)

Data are expressed as mean (±SD).

RO_max_ = Maximum average RO.

BRET signal arising from DY605-CTX and NLuc-EGFR interaction displayed a biphasic decay, with a rapid decay phase between 12 and 24 h followed by a slower decay phase between 24 and 168 h, whereas the NLuc donor emission did not decrease over the course of the study (Figures 6A–6D). Interestingly, the BRET signal of DY605-CTX exhibits a distinct decay profile compared with plasma clearance kinetics (Figure 5), indicating the discrete kinetics between plasma concentrations and target binding in solid tumors. Similar terminal slopes (*λ*_Z_) of BRET profiles for the different doses were observed, *λ*_Z_ = 0.011 ± 0.002, 0.013 ± 0.006, and 0.010 ± 0.011 for 50, 8.5, and 1.0 mg/kg groups respectively. The BRET ratios for 1.0 mg/kg dose group decayed to levels comparable to those of IgG controls at 48 h post injection (p = 0.23). We also calculated the AUC of BRET ratios (BRET integral) and found that the total AUC_BRET_ does not increase in a dose-proportionate manner. A trend of AUC_BRET_ saturation is observed (Table 3).

The RO was determined by dividing the BRET ratios at each time point by the average of the five highest BRET ratios observed throughout the study (Equation 6, see Transparent Methods). All the five highest BRET ratios were observed in the 50 mg/kg dose group, either at 4 or at 12 h post injection. As shown in Figure 6F, the ROs of three groups showed a trend similar to the BRET versus time curve (Figures 6F and 6E). The maximum average ROs were 72% ± 26%, 41% ± 14%, and 18% ± 6.0% at doses of 50, 8.5, and 1.0 mg/kg, respectively (Table 3), suggesting fractional target accessibility. Similar to the AUC_BRET_, the AUC_RO_ did not increase in a dose-proportional manner. The dose-normalized AUC_RO_ were 612 ± 197, 2,304 ± 674, and 6,417 ± 2600%·h/nmol for doses of 50, 8.5, and 1.0 mg/kg, respectively. Consistent with target binding versus time curves (Figure 6E), the RO curves declined in a biphasic manner, even though the plasma concentrations declined in a nearly mono-exponential manner between 12 and 168 h. A strong kinetics discrepancy was suggested between the systemic PK and RO in tumors.

### Dose-Exposure-RO Relationships

**Figure 7:**
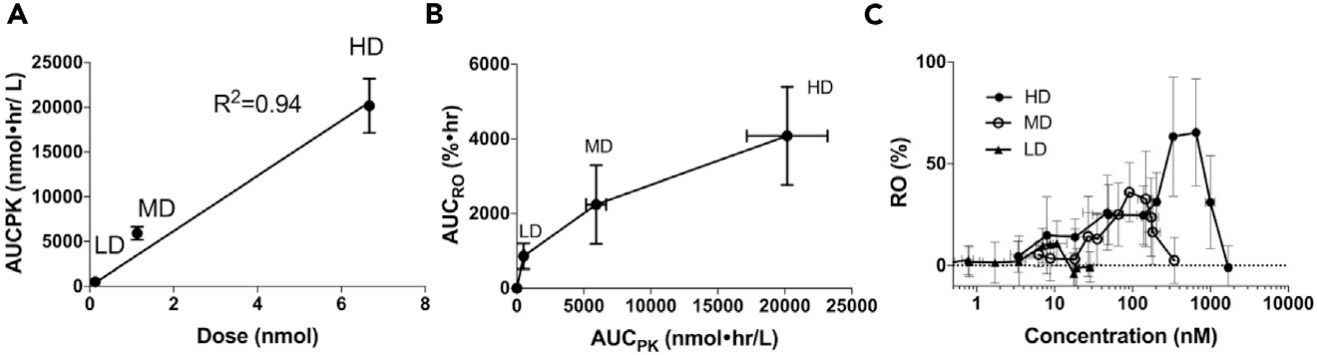
Direct Assessment of Dose-Exposure-Receptor Occupancy (RO) Relationships (A) Drug conjugate doses and plasma exposure (AUC_PK_) exhibited a linear relationship (R^2^ = 0.94). (B) AUC_PK_ and the area under the curve of RO (AUC_RO_) showed a sigmoidal relationship, suggesting a discontinuity between system exposure and RO in the tumor. For panels (A and B), each data point represents the mean of AUC_PK_ or AUC_RO_ values (n = 5). Error bars represent ±SD. The linear trends between AUC_PK_ and dose and between AUC_RO_ and AUC_PK_ were evaluated by nonlinear regression. (C) Relationships between RO and plasma concentrations. Hysteresis loops were observed in all dosed groups, indicating a temporal delay between plasma concentrations and ROs in the tumor.

The relationships between the dose and system exposure and between the system exposure and tumor RO were investigated. As shown in Table 2, DY605-CTX exhibited linear PK in the studied dose ranges (1.0–50 mg/kg), which was consistent with the dose-AUC_PK_ plot, as shown in Figure 7A. A non-linear relationship was observed between the AUC_RO_ and the AUC_PK_ (Figure 7B), indicating the discontinuity between systemic exposure and specific RO at tumor sites. The point-to-point relationships between the RO and drug plasma concentrations were described by hysteresis loops (Figure 7C), suggesting that the tumor ROs were not synchronized with plasma concentrations with a significant delay (several hours) existing between peak plasma concentration and maximal tumor RO.

## Discussion

Target engagement is a critical factor for the successful development of therapeutic antibodies, yet direct evidence of antibody-target interactions can be difficult to reproducibly achieve *in vivo* (Muller and Brennan, 2009). Many state-of-the-art tools have been applied to quantify RO. In FCM assays, RO is assessed by probing the ligand or receptors on circulating cells to provide evidence of sufficient target engagement. Despite its high sensitivity when assessing RO on circulating cells, FCM is not an ideal approach to assess RO in solid tumors. Solid tumors are highly heterogeneous, and the homogenization procedures required for FCM analysis disrupt tumor integrity, cause a loss of intratumoral spatial resolution, and compromise the overall accuracy of RO quantification if applied to solid tumors (Samkoe et al., 2014). In addition, the current methods immunohistochemistry and FCM for antibody target engagement in solid tumors require invasive procedures to obtain the necessary tissue biopsies. In this regard non-invasive whole-animal imaging approaches provide significant advantages in assessing the antibody-target interactions. However, commonly used displacement approaches for non-invasive RO assessment, in which small doses of radiotracer are replaced by increasing doses of unlabeled antibodies, are often complicated by cellular endocytosis and local turnover of the radiotracer. Non-invasive imaging approaches based on fluorescence are limited by high autofluorescence and fluorescence quenching (Samkoe et al., 2014). One common drawback of these imaging approaches is the lack of a signal specificity toward direct target engagement, which means that these imaging approaches cannot distinguish the signals of bound antibodies from those of free ones sequestered in the tissue of interest, or those bound non-specifically to non-target cells (endothelial, stromal, or other tumor associated cell types), resulting in a poor estimation of RO (Cunningham et al., 2005, Patel and Gibson, 2008, Ogawa et al., 2009, Eigenmann et al., 2017).

In the present study, we developed a BRET system that directly visualizes antibody-target interactions in live animals. In this BRET system, the bound DY605-CTX is triggered by substrate-dependent activation of NLuc (fused to EGFR), whereby any BRET emission reflects direct CTX-EGFR binding (Figure 1). Compared with conventional fluorescent imaging methods, the designed BRET method does not need external excitation light; thus the disadvantages of autofluorescence are avoided, whereas a high signal:ratio is promised. Despite limited spectrum overlap between NLuc emission (460 nm) and DY605 excitation (600 nm) (Figure S4), the high quantum output of NLuc is sufficient for triggering robust BRET (Hall et al., 2012, England et al., 2016, Alcobia et al., 2018). The long Stokes shift (165 nm) between DY605 emission peak (625 nm) and NLuc emission peak (460 nm) ensures robust spectral separation and enables the reliable detection of both the NLuc and the DY605 emission peaks. Mostly importantly, this BRET-based approach can be used to provide a direct measurement of antibody-target interaction in live animals. This observed BRET signal represents a real-time quantification of antibody-target complex. Thus because the derived RO is interpretable to several key physiologically relevant variables, such as antibody-antigen binding, antibody-antigen complex internalization, target turnover, and potential antibody-mediated trogo- or phagocytosis, RO measured by this approach could be used to directly compare the *in vivo* target engagement and therapeutic efficacy of different ligands for a given receptor (Weiner et al., 2010).

As expected, a temporal delay was observed between plasma concentrations of an antibody and its RO to the targets in tumors. The binding peaked in three DY605-CTX groups at about the same time after dosing (Figures 6A–6C and 6E), which suggested that the delay between systemic exposure and binding in the tumors was likely caused by a slow but linear diffusion process. Interestingly, this observation is inconsistent with those of the previous studies, which indicated dose-dependent tumor penetration (Graff and Wittrup, 2003, Rhoden and Wittrup, 2012). The reason for this inconsistency is unclear, but it might be associated with the quantification methods used in the present study or the relatively uniform vascularization in our xenograft models, evidenced by the lack of distribution-void necrotic area. High vascularization does not necessarily mean a full RO. As a matter of fact, only about 70%–80% of RO was achieved at the highest dose in the present study (Table 3). The fractional target binding could be caused by a mechanism called “binding site barrier” (Fujimori et al., 1990), which hypothesizes that the antibody-antigen complexes in the immediate proximity of blood vessels significantly decrease the penetration of free antibodies into deeper tumor tissue.

Interestingly, the CTX-EGFR binding in the present study exhibited a biphasic decline after peaking (12–168 h), whereas the PK was largely mono-exponential within the same observation period. This is in contrast to the linear decline of pharmacodynamics in Levy's “direct effect model” (Levy, 1966). The kinetic disassociation between antibody system exposure and target binding at tumor sites well supports our notion that plasma kinetics is not reflective of RO for antibodies that have targets in peripheral tissues (Thurber et al., 2008, Cao et al., 2013, Cao and Jusko, 2014). In addition, the target engagement level (AUC_RO_) did not increase in a dose-proportional fashion (Figure 7B). Comparing doses at 8.5 and 50 mg/kg, the average AUC_RO_ increased approximately twice. This non-dose-proportional increase of the AUC_RO_ and the average RO_max_ may be explained by the fractional target accessibility. Although the receptors were highly expressed in our system, as shown in *in vitro* results (Figures 2A and 2B), only a fraction of receptors were “accessible” to the antibodies, leading to a fractional RO, which was consistent with a previous study (Freeman et al., 2012), in which a high dose of panitumumab was assessed in A431-derived xenografts and only a small fraction of EGFR was occupied.

Overall, the present study provides a novel paradigm for selectively and directly monitoring antibody target engagement in live animals using a non-invasive approach. The non-invasive imaging technique described in this study could be utilized to establish solid tumor dose-RO-response relationships of mAbs that are critical in evaluating their therapeutic efficacies and support further exploration of the factors that affect mAb efficacy and toxicity.

### Limitations of the Study

Although the developed approach demonstrated feasibility in directly assessing RO in live animals, the approach has several technical limitations. First, we used the average of the five highest BRET signals to define 100% RO. Because of the lack of *in vivo* calibration curves, all derived ROs in the study are relative values rather than absolute quantifications. Although a true “100% RO” measurement cannot be quantified, the BRET-based system described herein provides a rapid and cost-effective way to evaluate “saturating RO” concentrations/dosing for any given drug-target pair in live animals in a temporally trackable format. Second, although the long Stokes shift (165 nm) limited the signal leakage from NLuc emission to DY605 emission, the background of the *in vivo* BRET measurement, that is the BRET ratios of DY605-IgG, was not zero. The highest signal:noise ratio observed in the quantified BRET ratio was approximately 4.2. This ∼20% background impaired the sensitivity of the RO assessment. Third, BRET ratios were quantified with inherent, protocol-constrained biases because of the restrictions on signal decay and filter settings. Owing to the intrinsic limitations of the imaging system, images at 500 and 620 nm could not be acquired simultaneously. Because of signal decay, the measurements at 500 nm were lower than the maximum potential peak value. In addition, the NLuc emission (460 nm) was collected using a non-optimal 500/20-nm band-pass filter, leading to an underestimation of donor emission signals. Fourth, the complex mixture of tumor and stroma cells in a given tumor microenvironment may further influence the BRET signals that can be achieved. Even though a non-homogenous cell distribution (as evidenced by a non-homogenous NLuc signal) in HEK293 xenograft (Figures 4E and 6A–6C) was observed, it may fail to capture the microenvironment present in “real” tumors, which may contain strong stromal and/or immune components. Therefore further evaluations of our approach in representative tumor models are warranted.

Although we acknowledge that these results are incremental in nature, our proof-of-concept study represents an extended use of BRET-based strategies toward mAb-target interactions, demonstrating that the mAb-dye conjugates retain the mAb PK features and the binding specificity and affinity to mAb's cognate antigens. Those features made BRET-based imaging methods amenable to determine both spatial and temporal kinetics of mAb-target interactions. Although the study by Alcobia et al. demonstrated that small molecule (ligands) can be labeled and utilized for BRET visualization, we demonstrate that this approach can be extended to mAbs, further bolstering their initial finding and extending the utility and applicability of BRET-based *in vivo* RO observation method broadly toward clinically viable and attractive targeted therapies.

## Methods

All methods can be found in the accompanying Transparent Methods supplemental file.

## Supplemental Information

Document S1. Transparent Methods and Figures S1–S4
