# Supplementary material for: A Bioluminescence Resonance Energy Transfer-Based Approach for Determining Antibody-Receptor Occupancy In Vivo
Source: iScience. 2019 May 8;15:439–51. doi: 10.1016/j.isci.2019.05.003 (PMC6529791; doi:10.1016/j.isci.2019.05.003)
Supplement: Document S1. Transparent Methods and Figures S1–S4 [file mmc1.pdf]

ISCI, Volume *15*

Supplemental Information

A Bioluminescence Resonance Energy Transfer-Based Approach for Determining Antibody-Receptor Occupancy *In Vivo*

Yu Tang, Kshitij Parag-Sharma, Antonio L. Amelio, and Yanguang Cao

Supplemental Information

A Bioluminescence Resonance Energy Transfer (BRET) Based Approach for Determining Antibody-Receptor Occupancy *In Vivo*

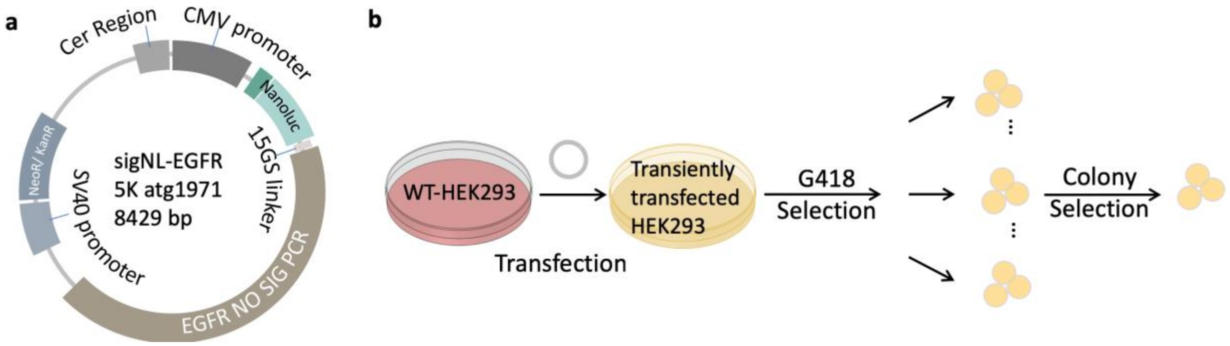

Figure S1. **Overview of NanoLuc-EGFR plasmid and selection scheme for generating the stable NanoLuc-EGFR HEK293 cells**, **Related to Figure 2.** (**a**) The NanoLuc-EGFR plasmid was a kind gift from Promega. NanoLuc was expressed at the N-terminus of EGFR by linking NanoLuc gene to the full-length human EGFR gene by 15GS linker. CMV promoter was designed for target gene (NanoLuc-EGFR sequences) and SV40 promoter was for Kan/Neo resistance gene. The size of the plasmid was 8429 bp. (**b**) The NanoLuc-EGFR HEK293 cells selection scheme. Transfection of SigNL-EGFR plasmid was conducted using Lipofectamine^{TM} 3000 Transfection Reagent according to the manufacturer's protocol. Selection of stably transfected HEK293 cells expressing NanoLuc-EGFR was performed with 1000 µg/mL geneticin. Single cell colonies with the highest expression of reporter protein were selected by measuring NanoLuc activity in the presence of the NanoLuc substrate furimazine. EGFR = Epidermal Growth Factor Receptor.

Supplemental Information

A Bioluminescence Resonance Energy Transfer (BRET) Based Approach for Determining Antibody-Receptor Occupancy *In Vivo*

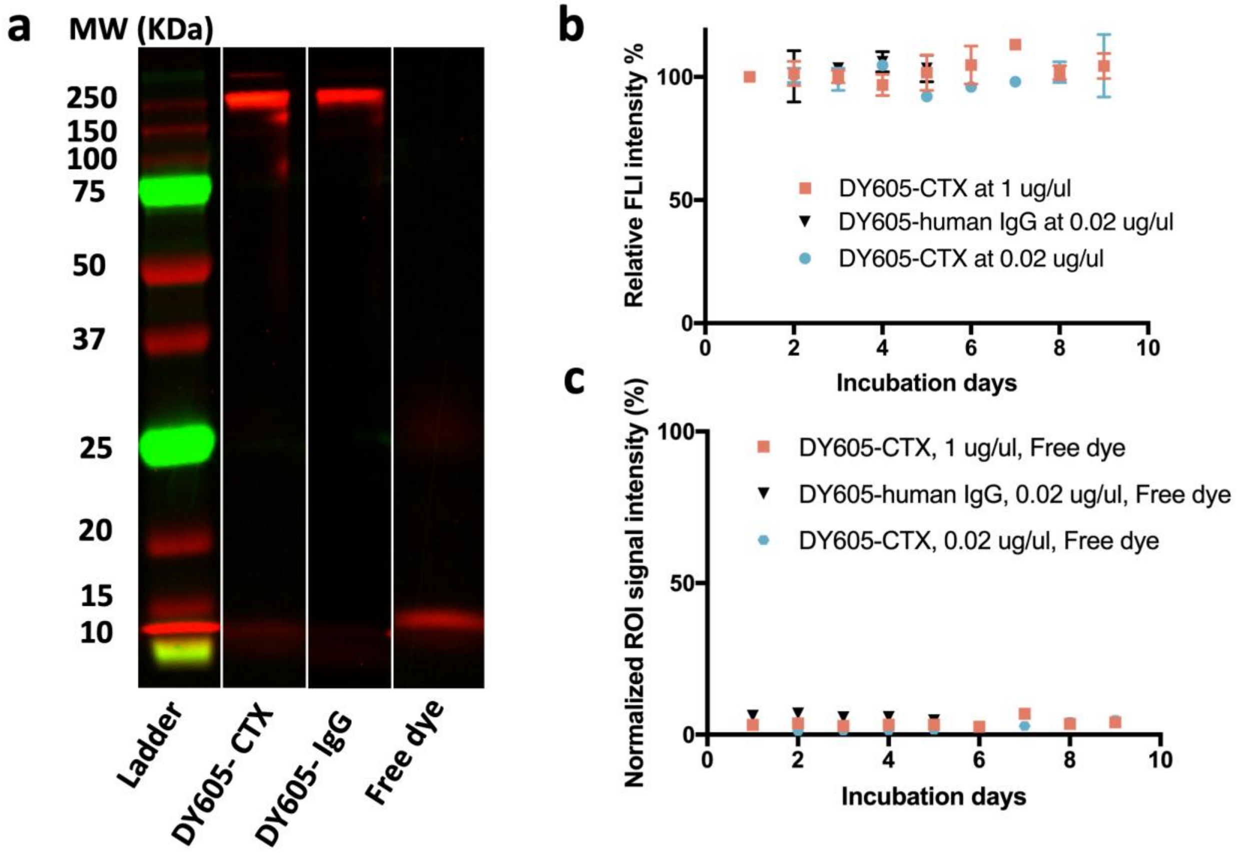

Figure S2. **Stability of DY605-cetuximab and DY605-IgG in mouse plasma**, **Related to Figure 5 and Figure 6.** (**a**) The residual free dye percent in DY605-cetuximab (3.6%) and DY605-IgG (3.3%) stock solution was determined by SDS-PAGE. The fluorescent intensities of the bands were quantified by ImageJ. (**b**) Total fluorescence of DY605-cetuximab and DY605-IgG mixture did not change during the incubation. The total fluorescence intensities of incubated conjugates-plasma solutions were normalized to the ones at day 1. The trend of total fluorescence was evaluated by testing the null hypothesis that “the slope of linear regression of the dataset was significantly non-zero”, which was rejected by a p value of 0.7. (**c**) No detectable conjugate dissembling was found in the DY605-cetuximab or the DY605-IgG throughout incubation. The ROI signal intensities of the free dye were normalized to the signal intensities of antibody-dye conjugates with the same incubation time. IgG = human IgG.

Supplemental Information

A Bioluminescence Resonance Energy Transfer (BRET) Based Approach for Determining Antibody-Receptor Occupancy *In Vivo*

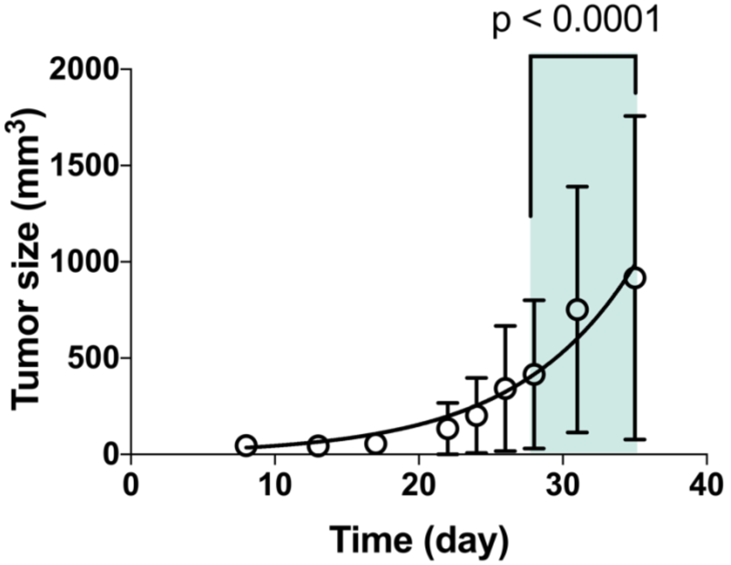

Figure S3. Caliper measurements quantifying tumor growth of NanoLuc-EGFR HEK293 xenografts, Related to Figure 4. Subcutaneous inoculation was performed on day 0. Palpable tumors were observed by day 8 – 13. The *in vivo* receptor occupancy detection phase was day 28 – 35, as denoted by green shadow. The tumor sizes at the end of *in vivo* RO detection study were significantly greater that the beginning of the study (two tailed, unpaired Students *t* test, $p < 0.0001$). The tumor growth was fitted to an exponential growth curve ($R^2 = 0.32$). Each data point represents the mean tumor size of 2 – 19 subjects. Error bars represent ±SD.

Supplemental Information

A Bioluminescence Resonance Energy Transfer (BRET) Based Approach for Determining Antibody-Receptor Occupancy *In Vivo*

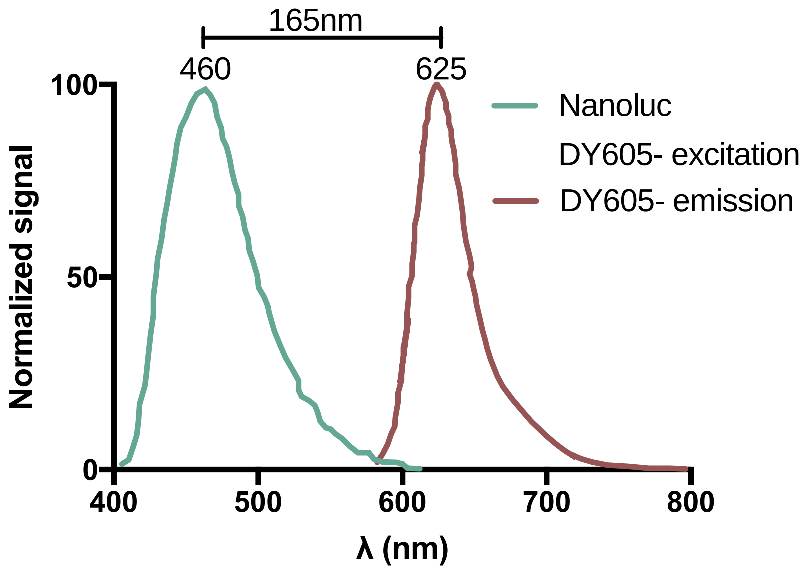

Figure S4. Profiles of normalized spectral scans for NanoLuc emission, DY605 excitation, and DY605 emission. Related to Figure 1.

TRANSPARENT METHODS

Design of the NanoLuc-EGFR / DY605-cetuximab BRET imaging system

The SigNL-EGFR plasmid, containing the NanoLuc luciferase fused to the N-terminus of EGFR, was a kind gift from Promega Life Science (Seattle, WA, USA). The plasmid map is shown in **Fig. S1a**. A hydrophilic fluorescent dye, DY605 (Dyomics GmbH, Jena, Germany), was selected as the BRET acceptor because of its outstanding molar absorbance (110,000 $M^{-1}cm^{-1}$) and spectrum ($\lambda_{ex/em}$ = 600/625 nm). DY605 was covalently appended onto the lysine residues of cetuximab (Erbitux, Eli Lilly) using *N*-hydroxysuccinimide (NHS) chemistry to generate the DY605-cetuximab (DY605-CTX). Briefly, the pH of the cetuximab solution was adjusted to 8.0−8.5 by using a solution of 7.5% sodium bicarbonate (Gibco®, Gaithersburg, MD, USA) before the DY605 stocking solution (13.3 nmol/μl) was added. The mixture of DY605 and cetuximab was blended at room temperature for 1 hr. Lysines are a common site for nonspecific conjugation of fluorescent dyes using ester chemistry (Cilliers et al., 2017). There are 44 lysines on cetuximab, about 3/4 of which are on the heavy chain (Wishart et al., 2006). As conjugation between cetuximab and DY605 is nonspecific, most of DY605 is expected to be appended on heavy chain of cetuximab. After conjugation, free dye was removed using desalting columns from GE Healthcare (Piscataway, NJ, USA). Antibody concentrations and dye/antibody ratios (DARs) were measured using Nanodrop 1000 (Wilmington, DE, USA) as the equations below. DY650 was appended to IgG (control) using an identical protocol.

$$\text{Antibody concentration (mg/mL)} = \frac{\text{A280}-\text{A600}\cdot\text{Correction Factor}}{1.4} \qquad \text{Eq. 1}$$

The antibody concentration was estimated by assuming 1.4 $A_{protein}$ units = 1 mg/mL (for IgG). Correction Factor for DY605 = 0.552.

$$\text{DAR} = \frac{\text{A600}\cdot\text{antibody molecular weight (g/mol)}}{\text{antibody concentration (mg/mL)}\cdot\text{molar absorbance (M}-1\text{ cm}-1)} \qquad \text{Eq. 2}$$

Cetuximab molecular weight = 150, 000 g/mol. DY605 molar absorbance = 110,000M^{-1}. For the study evaluating the DAR effect on the DY605-CTX pharmacokinetics (PK), cetuximab was labeled with DY605 at three DARs (1.6, 5.9, and 13) for comparison. The DARs were tuned by changing the loading amount of DY605 when conjugating to cetuximab.

The schematic of how this BRET system works is illustrated in Fig. 1. In the absence of DY605-CTX binding, the addition of the NanoLuc (NLuc) substrate (furimazine) results in the single emission peak at 460nm of NLuc (donor) is observed (**Fig. 1A**). However, upon DY605-CTX binding to NLuc-EGFR (bringing NLuc into close proximity with the DY605 fluorophore), the addition of furimazine produces two distinct peaks, at 460nm (NLuc) and at 625 (emission peak of DY605), the latter arising from the robust BRET observed between NLuc and DY605 (**Fig. 1B**). Both donor (at 460nm) and acceptor (at 625nm) emission peaks are ~165nm apart ensuring robust spectral separation and reliable detection (**Fig. S4**).

Cell culture, transfection, and clonal Isolation

The cell model was developed on wild type HEK293 cell line (WT HEK293) due to its intrinsically low EGFR expression (Zhang et al., 2015). HEK293 cell line was obtained from UNC tissue culture facility. HEK293 cells were cultured in Dulbecco's Modified Eagle Medium (Gibco®, Gaithersburg, MD, USA) containing 1% penicillin/streptomycin supplemented (Gibco®, Gaithersburg, MD, USA) with 10% fetal bovine plasma (EMD Millipore, Burlington, MA, USA). Prior to transfection, WT HEK293 cells were seeded in 12-well plates and cultured in complete growth medium. The transfection of the SigNL-EGFR plasmid was conducted using Lipofectamine™ 3000 Transfection Reagent (Invitrogen; Carlsbad, CA, USA) according to the manufacturer's protocol. Selection of stably transfected HEK293 cells expressing NLuc-EGFR was performed with 1000 μg/mL geneticin (Invitrogen; Carlsbad, CA, USA). Single cell colonies with the highest expression of reporter protein were selected by measuring the NLuc activity in the presence of the NLuc substrate furimazine (Promega, Madison, WI, USA). NLuc activities of wildtype versus NLuc-EGFR expressing cells (suspensions) were compared under identical conditions (same cell density and concentrations of the furimazine substrate). Furimazine was added to cells according to manufacturer's protocols (25 μL per well after a 20-fold dilution). The NLuc activity was measured using Cytation 3 (Biotek; Winooski, VT, USA) using a 460/40 nm bandpass filter set at room temperature. The clone selection

scheme was summarized in **Fig. S1b**. These stably transfected cells exhibiting the highest NLuc luminescence were used in all subsequent experiments.

NLuc-EGFR expression was also evaluated using anti-EGFR antibody probing (DY605 labeled cetuximab, DAR = 3.8). NLuc-EGFR HEK293 cells and WT HEK293 cells were suspended in Opti-MEM at equal cell densities and incubated with 100 nM of either DY605-CTX or DY605-IgG for 1 hr at 4 °C. After the incubation, cells were recovered, spun down, washed three times using cold Dulbecco's phosphate-buffered saline (Gibco®, Gaithersburg, MD, USA) and then resuspended in Opti-MEM (Gibco®, Gaithersburg, MD, USA). The cell suspension was transferred to black 96-well plates (Corning Inc. Corning, NY, USA), equalized for volume. The fluorescence was quantified using Cytation 3 fluorescence monochromator with 16 nm bandwidth at $\lambda_{ex/em}$ = 580/610 nm with a gain of 100. For both NLuc activity evaluation and EGFR probing, at least 5 technical replicates were included in one experimental group. The experiments were repeated at least three times.

In vitro **BRET assay**

CTX-EGFR binding was quantified using BRET measurements. Briefly, NLuc-EGFR HEK293 cells were seeded in white 96-well plates 24 hr before the experiments. Complete medium in 96-well plates was then replaced by Opti-MEM 1 hr prior to the experiments. A dose titration of DY605-CTX or DY605-IgG was performed in the presence/absence of 1 mM unlabeled cetuximab. After incubation for 1 hr at room temperature, the NLuc substrate furimazine was added to each well following manufacturer's instructions (25 μL per well after a 20-fold dilution). The luminescence was measured at 460 nm and 625 nm by Cytation 3 equipped with 460/40 nm bandpass filter and 610 nm longpass filter. The BRET ratio was calculated by dividing the 625 nm emission by the 460 nm emission and was corrected using the baseline BRET ratio, as Eq. 3 shows.

$$\text{BRET ratio } (\textit{in vitro}) = \frac{\text{Acceptor emission (sample)}}{\text{Donor emission (sample)}} - \frac{\text{Acceptor emission (Blank)}}{\text{Donor emission (Blank)}} \qquad \text{Eq.3}$$

Three technical replicates were included in the *in vitro* BRET assay. The experiments were repeated at least three times.

Effect of DARs on target binding and pharmacokinetics (PK) of DY605-CTX in mice

Antibody PK alterations due to fluorophore types or DAR have been reported (Boswell et al., 2012; Zhang et al., 2016; Cilliers et al., 2017). While increased DAR elevates the intensity of BRET acceptor emissions, it has been shown to accelerate the clearance of dye-antibody conjugates (Cilliers et al., 2017). In the present study, the effect of DARs on PK was investigated, prior to the *in vivo* receptor occupancy (RO) study. We labeled cetuximab with DY605 at three different DARs (1.6, 5.9, and 13). The filtered DY605-CTX (DAR= 1.6, 5.9, and 13) were diluted in saline to 0.64 mg/mL for the following PK study in mice.

Female nude mice were obtained from the Division of Comparative Medicine at UNC. Each mouse was injected with 100 µl of DY605-CTX saline solution via tail vein (3.2 mg/kg, n = 3/DAR group). Blood samples were collected in a rotating manner within three subsets per group, and the total amount of blood removed from each subgroup was kept consistent throughout the study. Blood samples (30 µl) were collected via tail vein at 0, 18, 24, 48, 72, 96, 120, and 168 hr post dosing. Plasma fluorescence was measured immediately after sampling and the rest of the sample was snap frozen and stored at -80°C. Plasma concentrations of DY605-CTX were measured based on fluorescent intensities according calibration curves. All animal studies were conducted in compliance with the Institutional Animal Care and Use Committee (IACUC) of UNC.

Stability of DY605-CTX in mouse plasma

The conjugate stability was tested as previously described (Aldrich et al., 2011; Cilliers et al., 2017). Briefly, DY605-CTX was added into mouse plasma at the final concentrations of 1 µg/µl or 0.02 µg/µl. Plasma aliquots were stored at –80°C, then thawed and incubated at 37°C for 9 days for DY605-CTX, 5 days for DY605 -IgG. The mixtures were thawed in reverse order, namely, the one thawed on the 9th day served as the first time point (day 1) of DY605-CTX, so that all samples were measured on the same day.

After incubation, 2 µl of either the DY605-CTX or the DY605-IgG plasma samples were mixed with 200 µl of saline in each well in a 96-well plate. The total fluorescence of incubated conjugates was measured by Cytation 3 (Biotek; Winooski, VT, USA) and normalized to the ones at day 1. The conjugate-specific fluorescence was determined after SDS-PAGE separation. Specifically, the DY605-CTX or the DY605-IgG plasma samples were mixed with LaemmLi buffer (Bio-Rad; Hercules, CA, USA) at 0.33 mg/mL and heated at 95℃ for 5 min. The conjugate solutions were loaded on 12% polyacrylamide gel (Bio-Rad; Hercules, CA, USA), free dye was used as a control. The electrophoresis was performed in Tris/HEPES/SDS running buffer (Bio-Rad; Hercules, CA, USA) for 45 min at 100 volts. Immediately after electrophoresis, the gels were removed and scanned with FluorChem M (Cell Biosciences, Inc., Santa Clara, USA). The fluorescent intensities of the DY605-CTX, DY605-IgG, and free dye bands were quantified using ImageJ (National Institutes of Health, Bethesda, MD). The ROI signal intensities of the free dye were normalized to the signal intensities of antibody-dye conjugates with the same incubation time.

Assessment of RO in live mice

A mouse xenograft model of NLuc-EGFR HEK293 was established using female nude mice (4 - 6 weeks). To establish tumor models, 5×10^6 NLuc-EGFR HEK293 cells were suspended in 0.1 mL of PBS/ Matrigel (BD Biosciences; San Jose, CA, USA) (1/1, v/v) and inoculated subcutaneously into the inguinal flank of the nude mice. After ~4 weeks, mice with similar tumor sizes (> 500 mm^3) were selected for the imaging experiments.

Tumor growth was measured using calipers every other day. Tumor volumes were calculated according to the equation below.

$$\text{Tumor size (mm}^3) = \frac{\text{length (mm)}\cdot\text{weidth (mm)}\cdot\text{weidth(mm)}}{2} \qquad \text{Eq. 4}$$

Once the tumor sizes surpassed 500 mm^3, the mice were randomly assigned to 4 groups: control (DY605-IgG at 1.9 mg/kg) or 3 treatment groups for DY605-CTX at 1.0, 8.5, and 50 mg/kg (n = 5/dose). The DY605-CTX (DAR = 4.6) and DY605-IgG (DAR = 5.6) were administered via tail vein injection. Animals were anesthetized and fixed to prevent moving during the image acquisition. Prior to image acquisition, furimazine (0.25 mg/kg) was injected via tail vein. Images were acquired at time 0, 2, 4, 6, 12, 24, 48, 72, 96, 120, 144, and 168 hr after the injection of either DY605-CTX or DY605-IgG. The images were acquired around 45 s after substrate administration with the same order (620 nm first). The parameters used during the imaging were, 1 min exposure time (luminescent f/stop = 1) with either 620/20 nm or 500/20 nm bandpass filter set. All the *in vivo* images were acquired using an IVIS Kinetic optical imaging system (Caliper Life Sciences, Alameda, CA, USA) that was equipped with an electron multiplying charge-coupled device camera.

Acquired images were processed and quantified using Living image 4.5.2 (Caliper Life Sciences, Alameda, CA, USA). For tumor size and receptor density analysis, the whole tumor area was gated and the total flux at tumor area was measured. The signal intensity was quantified using the total flux and the average radiance, which reflected the total NLuc-EGFR HEK293 amount and density, respectively. For RO quantification, up to 6 non-overlapped regions of interest were gated at the tumor areas on each image at 620/20 nm and 500/20 nm. To keep consistency, the sizes and locations of all ROIs for each tumor were the same at each time point. The total flux of all ROIs on corresponding acceptor/donor images were quantified. The average DY605-IgG BRET ratio that was measured within the first 48 hrs post administration was used as background, which was subtracted from raw BRET ratios in 50 mg/kg, 8.5 mg/ kg, and 1.0 mg/kg DY605-CTX group. The BRET ratio of a given ROI was calculated as the equation below.

$$\text{BRET ratio } (\textit{in vivo}) = \frac{\text{Total flux acceptor (DY605-CTX)}}{\text{Total flux donor (DY605-CTX)}} - \frac{\text{Total flux acceptor (DY605-IgG)}}{\text{Total flux donor (DY605-IgG)}} \qquad \text{Eq. 5}$$

In addition, at each time point, 30 µl blood samples were collected via tail vein for PK assessment. After the study, the animals were euthanized.

The five highest BRET ratios obtained throughout the *in vivo* RO assessment (DAR = 4.6; 50, 8.5, and 1.0 mg/kg dose groups, from 0 to 168 hr post injection) were selected and their average defined as RO = 100%. The remaining RO was calculated using the equation below.

$$\text{RO\%} = 100\% \cdot \frac{\text{BRET ratio (DY605-CTX)}}{\text{Highest BRET ratio (DY605-CTX)}} \qquad \text{Eq. 6}$$

Data analysis

The PK data was analyzed using Phoenix WinNonlin 7.0 (Certara, Princeton, NJ, USA) to calculate the PK parameters including maximum concentration (C_{max}), area under the curve ($AUC_{0\text{-}168hr}$), and clearance (CL). The max values, terminal slopes (λ_Z), and AUC of receptor binding and RO data were also calculated. All experimental findings were statistically evaluated using GraphPad Prism version 7.0d (GraphPad Software; La Jolla, CA, USA).

SUPPLEMENTAL REFERENCES

Aldrich MB, Wang X, Hart A, Kwon S, Sampath L, Marshall MV and Sevick-Muraca EM (2011) Assessment of free dye in solutions of dual-labeled antibody conjugates for in vivo molecular imaging. *Mol Imaging Biol* **13**:32-42.

Boswell CA, Bumbaca D, Fielder PJ and Khawli LA (2012) Compartmental tissue distribution of antibody therapeutics: experimental approaches and interpretations. *AAPS J* **14**:612-618.

Cilliers C, Nessler I, Christodolu N and Thurber GM (2017) Tracking Antibody Distribution with Near-Infrared Fluorescent Dyes: Impact of Dye Structure and Degree of Labeling on Plasma Clearance. *Mol Pharm* **14**:1623-1633.

Dyomics (Spring 2017) Fluorescent dyes for bioanalytical and hightech applications, 8th edition.

Wishart DS, Knox C, Guo AC, Shrivastava S, Hassanali M, Stothard P, Chang Z and Woolsey J (2006) DrugBank: a comprehensive resource for in silico drug discovery and exploration. *Nucleic Acids Res* **34**:D668-672.

Zhang F, Wang S, Yin L, Yang Y, Guan Y, Wang W, Xu H and Tao N (2015) Quantification of epidermal growth factor receptor expression level and binding kinetics on cell surfaces by surface plasmon resonance imaging. *Anal Chem* **87**:9960-9965.

Zhang L, Navaratna T and Thurber GM (2016) A Helix-Stabilizing Linker Improves Subcutaneous Bioavailability of a Helical Peptide Independent of Linker Lipophilicity. *Bioconjug Chem* **27**:1663-1672.
